# Influence of Tranexamic Acid on Elution Characteristics and Compressive Strength of Antibiotic-Loaded PMMA-Bone Cement with Gentamicin

**DOI:** 10.3390/ma14195639

**Published:** 2021-09-28

**Authors:** Martin Lüdemann, Axel Jakuscheit, Andrea Ewald, Leena Frühmann, Stefanie Hölscher-Doht, Maximilian Rudert, Sebastian Philipp von Hertzberg-Boelch

**Affiliations:** 1 Department of Orthopaedic Surgery, University of Wuerzburg, Koenig-Ludwig-Haus, Brettreich-Str. 11, 97074 Wuerzburg, Germany; a-jakuscheit.klh@uni-wuerzburg.de (A.J.); leena.f@hotmail.de (L.F.); m-rudert.klh@uni-wuerzburg.de (M.R.); s-boelch.klh@uni-wuerzburg.de (S.P.v.H.-B.); 2 Department for Functional Materials in Medicine and Dentistry, University of Wuerzburg, Pleicherwall 2, 97070 Wuerzburg, Germany; andrea.ewald@fmz.uni-wuerzburg.de; 3 Department of Trauma-, Hand-, Plastic- and Reconstructive Surgery, University of Wuerzburg, Oberduerrbacher-Str. 6, 97080 Wuerzburg, Germany; Hoelscher_S@ukw.de

**Keywords:** gentamicin-loaded poly (methyl methacrylate) bone cement, total joint arthroplasty, total knee arthroplasty, tranexamic acid

## Abstract

Purpose: The topical application of tranexamic acid (TXA) into the joint space during total joint arthroplasty (TJA) with no increase of complications, has been widely reported. We investigated the influence of TXA on antibiotic release, activity of the released antibiotic against a clinical isolate of *S. aureus*, and compressive strength of a widely used commercially prepared gentamicin-loaded cement brand (PALACOS R + G). Method: 12 bone cement cylinders (diameter and height = 6 and 12 mm, respectively) were molded. After curing in air for at least 1 h, six of the cylinders were completely immersed in 5 mL of fetal calf serum (FCS) and the other six were completely immersed in a solution consisting of 4.9 mL of FCS and 0.1 mL (10 mg) of TXA. Gentamicin elution tests were performed over 7 d. Four hundred µL of the gentamicin eluate were taken every 24 h for the first 7 d without renewing the immersion fluid. The gentamicin concentration was determined in a clinical analyzer using a homogeny enzyme immuno-assay. The antimicrobial activity of the eluate, obtained after day 7, was tested. An agar diffusion test regime was used with *Staphylococcus aureus*. Bacteria were grown in a LB medium and plated on LB agar plates to get a bacterial lawn. Fifty µL of each eluate were pipetted on 12-mm diameter filter discs, which were placed in the middle of the agar gel. After 24 h of cultivation at 37 °C, the zone of inhibition (ZOI) for each specimen was measured. The compressive strength of the cements was determined per ISO 5833. Results: At each time point in the gentamicin release test, the difference in gentamicin concentration, obtained from specimens immersed in the FCS solution only and those immersed in the FCS + TXA solution was not significant (*p* = 0.055–0.522). The same trend was seen in each of the following parameters, after 7 d of immersion: (1) Cumulative gentamicin concentration (*p* < 0.297); (2) gentamicin activity against *S. aureus* (strongly visible); (3) ZOI size (mostly > 20 mm) (*p* = 0.631); and (4) compressive strength (*p* = 0.262). Conclusions: For the PALACOS R + G specimens, the addition of TXA to FCS does not produce significant decreases in gentamicin concentration, in the activity of the gentamicin eluate against a clinical isolate of *S. aureus*, the zone of inhibition of *S. aureus,* and in the compressive strength of the cement, after 7 d of immersion in the test solution.

## 1. Introduction

The poly (methyl methacrylate) (PMMA) bone cement has been widely used in orthopedic and traumatologic surgery since the 1940s. Charnley popularized the cement in the early 1960s as the fixation agent for total hip arthroplasty [1]. The ability of the PMMA bone cement to provide an immediate mechanical stability explains its many current uses in orthopedic surgery, which include arthroplasty, trauma, and tumor. A special feature is the prevention of infection in primary total knee arthroplasty (TKA). In addition, PMMA is increasingly used as a drug carrier for local antibiotic treatment in cases of skeletal infection [2]. Here, an antibiotic-loaded bone cement (ALBC) fills the dead space and provides high local levels of the antibiotic. The release of an antibiotic from an ALBC is a diffusion process, which is influenced by several factors such as the antibiotic used, antibiotic loading, specimen surface area, and specimen porosity [3]. Another application of ALBCs is for the anchoring of total joint arthroplasty (TJA) with gentamicin, tobramycin, and vancomycin, which are the antibiotics most often used. In fact, anchoring using an ALBC is now the standard of care in primary TKA. Data from joint registries demonstrate an improvement in the survivorship of TJA by reducing the incidence of both septic and aseptic failures [2,4,5]. However, a challenge with TJA is the considerable blood loss, which may lead to acute anemia and a series of complications, increasing the need for allogenic blood transfusions, which are expensive and have adverse effects [6,7]. It is known that tranexamic acid (TXA), a synthetic derivate of the amino acid, lysine, exerts an antifibrinolytic effect through the reversible blockade of lysine-binding sites on plasminogen molecules and interferes with fibrinolysis. TXA, applied intravenously or locally into the joint space during TJA, has been reported to reduce peri-operative blood loss, without the increased incidence of complications, such as pulmonary embolism, deep vein thrombosis, and wound infection [8,9]. We are not aware of any literature on the influence of TXA on the properties of ALBC. The purpose of the present work was to carry out this investigation, with reference to a commercially-prepared gentamicin-loaded PMMA bone cement brand, which is approved for clinical use by many regulatory bodies around the world. The cement properties determined were gentamicin release, activity of the released gentamicin against a clinical isolate of *S. aureus*, and compressive strength.

## 2. Materials and Methods

### 2.1. Materials

The ALBC brand used was PALACOS^®^ R + G (premixed 0.5 g of gentamicin per 40.8 g of powder, amounting to 1.34 wt./wt. of gentamicin) (Heraeus, Medical GmbH, Wehrheim, Germany).

### 2.2. Specimen Preparation

The method of the specimen preparation was detailed previously [10]. All the cements were mixed under atmospheric pressure using all of the powder and liquid (20 mL). Twelve cylindrical specimens (diameter and height = 6 and 12 mm, respectively) were molded as stipulated in ISO 5833 [11]. All the specimens were kept at room temperature for 1 h. Thereafter, six specimens were completely immersed in 5 mL of FCS (Bio&Sell, Feucht, Germany) in wells in a 12-well plate. The other six specimens were completely immersed in a solution consisting of 4.9 mL of FCS + 0.1 mL (10 mg) TXA (Cyklokapron^®^, Pfizer, Berlin, Germany) in wells in the plate.

### 2.3. Gentamicin Release Test

These tests were performed at room temperature. Four hundred µL of the eluate were taken every 24 h for the first 7 d without renewing the immersion fluid. In addition, the eluate was stored at −20 °C until the gentamicin concentration was determined in the eluate. The concentration was measured using a clinical analyzer (Hitachi Analyzer, Roche, Mannheim, Germany) with a homogeny enzyme immuno-assay (Online TDM Vancomycin Cobas Roche (lowest limit of measurement (LLM) = 1.7 µg/mL and CEDIA^®^ Gentamicin II Assay, Microgenics, Passau, Germany (LLM = 0.24 µg/mL)) [12].

### 2.4. Antimicrobial Activity Test

The antimicrobial activity of the eluted released gentamicin was determined using an agar diffusion test regime with a Gram-positive bacterium, *Staphylococcus aureus*, clinically isolated, as described before [13]. Bacteria were grown in a LB medium (2 g yeast extract, 4 g tryptone (both Applichem GmbH Darmstadt, Germany, 2 g NaCl (Sigma-Aldrich, Darmstadt, Germany) and 400 mL H_2_O double dest)) and plated on LB agar plates (LB medium containing 1.5% (*w/v*) agar (Applichem GmbH, Darmstadt, Germany)) to get a bacterial lawn. Fifty µL of the eluate were pipetted on filter discs (diameter = 12 mm), which were placed in the middle of the agar gel. After 24 h of cultivation at 37 °C, the extent of the inhibition zones was measured.

### 2.5. Compression Test

After immersion for 7 d in the test fluid, the compression strength of the cements was determined using a servohydraulically-actuated material testing machine (Z020, Zwick/Roell, Ulm, Germany) and the test-Xpert software (version 3.6, Zwick/Roell, Ulm, Germany). A 20 kN load cell was connected to the indenter (radius = 6 mm) to apply a monoaxial compression at 10 mm/min to the specimen. The test was performed up to a maximum of 20 kN. From the load-displacement curve obtained, the compressive strength was determined following the steps detailed in ISO 5833.

### 2.6. Statistical Analysis

All the results are presented as the mean ± standard deviation. To test for normality of a population, the Kolmogorov-Smirnov test was used. In the case of normal distribution, a comparison between the means of the populations was conducted using the *t*-test for unpaired samples. For a distribution that was found not to be normal, the test for difference in means was conducted using the Mann-Whitney U test. Significance was indicated if *p* < 0.05. All the tests were performed using a commercially-available software package (SPSS 25.0; SSPS, Inc., Chicago, IL, USA).

## 3. Results

### 3.1. Released Gentamicin Concentration

**Figure 1:**
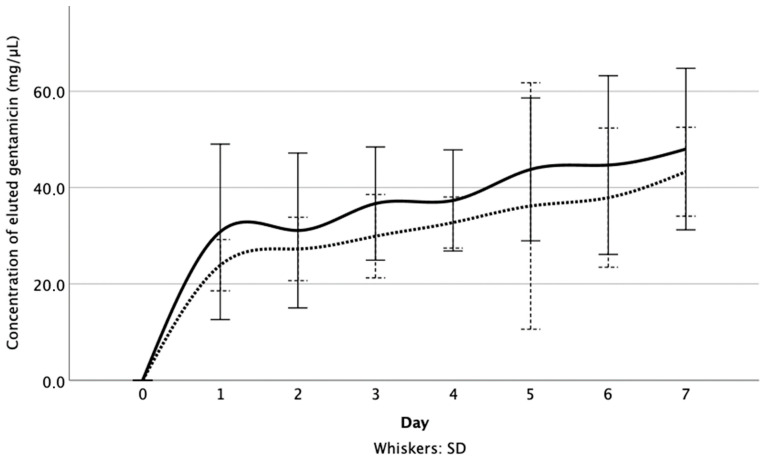
Gentamicin concentration [µg/mL] for gentamicin-loaded PMMA with FCS and tranexamic acid admixture (group 2—broken line) vs. FCS only (group 1—continuous line), all of the measurements are shown in both groups.

**Figure 2:**
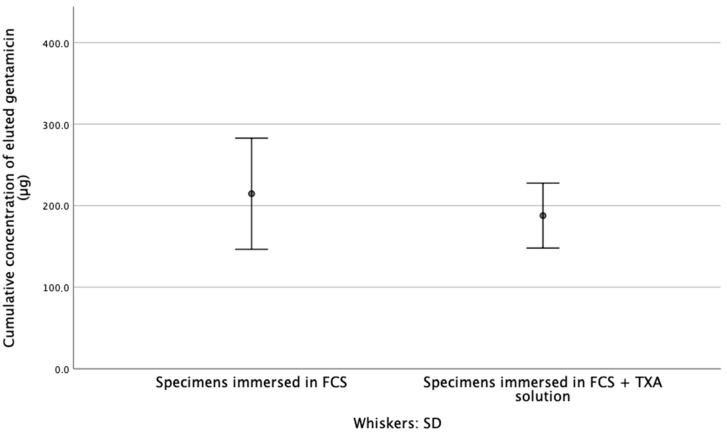
Summary of cumulative released gentamicin concentration results after 7 d of the elution test.

At each time point, the difference in the concentration of gentamicin released from the test specimens was not significant (*p*_1,3,5_ = 0.055, *p*_2_ = 0.423, *p*_4,6_ = 0.200, *p*_7_ = 0.522) (Figure 1). Furthermore, after immersion for 7 d, the cumulative concentration of the released gentamicin, from specimens that were immersed in FCS (216 ± 13.92 µg) was not significantly higher than that from specimens that were immersed in the FCS + TXA solution (187 ± 8.14 µg) (*p* = 0.297) (Figure 2).

### 3.2. Zone of Inhibition

**Figure 3:**
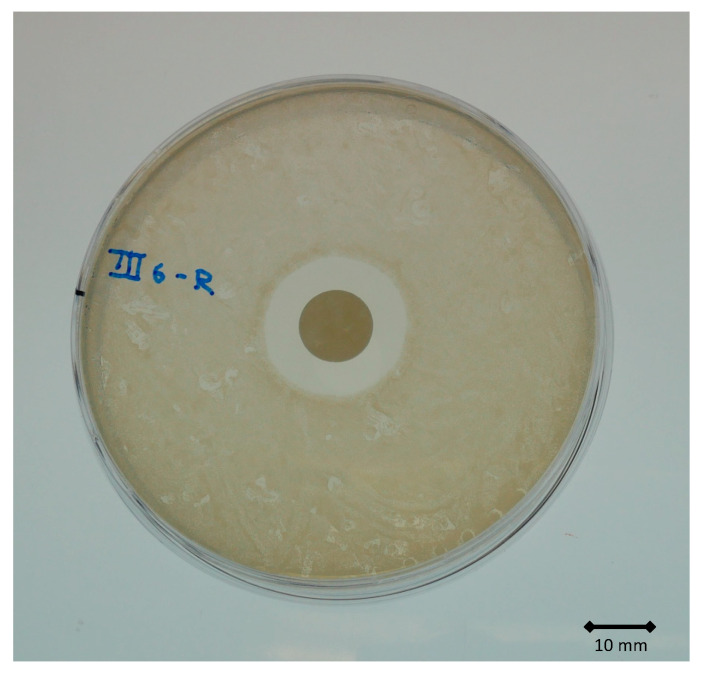
Zone of inhibition of the eluted gentamicin from the specimen, after 7 d of immersion in the test solution, against a clinical isolate of *S. aureus*.

**Figure 4:**
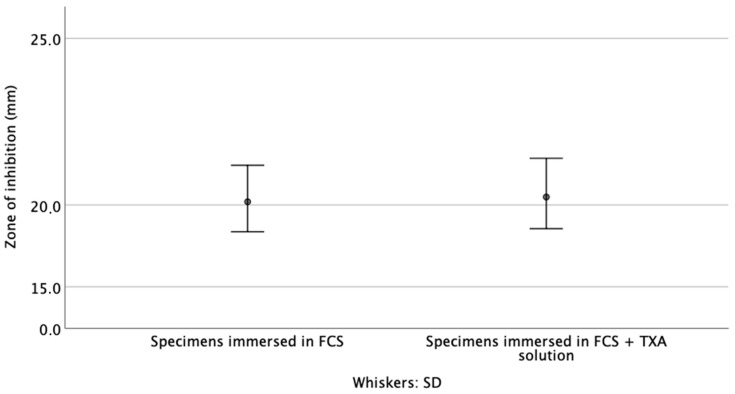
Summary of the zone of inhibition of the cements, after 7 d of immersion in the test solution.

After 7 d of immersion in either FCS or FCS + TAX solution, gentamicin was still highly active, with clearly visible ZOI > 20 mm (Figure 3). At this timepoint, the difference in ZOI between the two sets of specimens was not significant (*p* = 0.631) (Figure 4).

### 3.3. Compressive Strength

**Figure 5:**
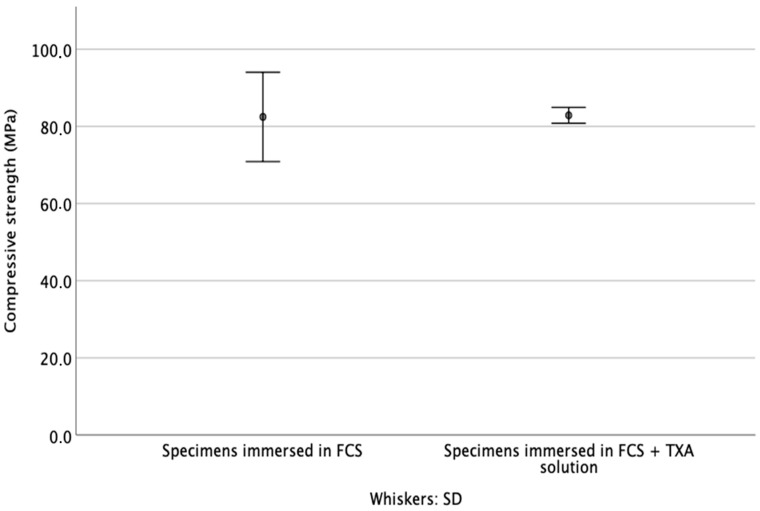
Summary of the compressive strength of the cements, after 7 d of immersion in the test fluids.

After 7 d of immersion in the test fluid, the difference in the compressive strength of the cements in study groups 1 and 2 was not significant (*p* = 0.262) (Figure 5).

## 4. Discussion

Anchoring the prostheses in a bed of ALBC is now the standard of care for primary TJA, since this provides mechanical stability and high local antibiotic concentrations for the prevention of infection of the prostheses [2,5,14]. The topical or intravenous application of TXA is widely used for reducing blood loss after TJA [8,9,15]. It is well known that several endogenous and exogenous variables influence polymerization kinetics, mechanical properties, and other aspects of the performance of ALBCs, for example, these variables include storage conditions prior to mixing and the method of mixing the antibiotic with the cement powder [3,16,17]. We know of no prior study on the influence of the topical application of TXA into the joint space during TJA on any properties of the cement. In the present study, we showed that the topical application of TXA into the joint during TJA using a gentamicin-loaded PMMA bone cement does not significantly reduce the elution of gentamicin from the cement. Many variables influence the release of an antibiotic from an ALBC, in particular cement composition, specimen surface area, and porosity [3]. The present results suggest that none of these variables is significantly affected by TXA. In a meta-analysis by Li et al., the excellent clinical efficacy and safety were found with the topical administration of 1–3 g TXA in patients who underwent primary total knee and hip arthroplasties [18]. Moreover, no adverse effects, such as pulmonary embolism, deep vein thrombosis or superficial infection, were associated with this administration [18]. Boelch et al. reported on the influence of eluate volume on the concentration of antibiotics released from the ALBC specimens [19]. In the present work, the cement specimens were immersed in 5 mL of fluid (0.1 mL TXA + 4.9 mL FCS or FCs) for 7 d. In each of the test fluids (FCS + TXA and FCS only), our elution test results showed that the gentamicin release profile was a diffusion process, with the maximum release taking place during the first day of the test, which is the period when the gradient between the carrier and its surrounding is at its highest. The TXA proportion in the FCS solution corresponds to 1 g of TXA in the clinical application, considering a postoperative wound drainage volume of 500 mL into the joint spacer [19]. This dosage is compatible with the clinical practice guidelines for a low dose topical application during TJA [20]. We suggest that in vivo, the gentamicin release profile will be very similar. Another issue is how long TXA acts after the topical application. A biological half-life of 2–3 h within the joint fluid was reported, whereas in the case of intra-articular TXA application, the acid enters the extravascular tissue space and accumulates up to 17 h. For the in vitro setting without formation of the plasminogen complex and without resorption, TXA is not degraded [21]. The influence of TXA on the measurement of the antibiotic concentration using an enzyme immuno-assay system is unclear. On the one hand, sisomicin, which similar to gentamicin, is an aminoglycoside, showed a significant interference (62.10%), but other antibiotics, including other aminoglycosides, such as amikacin, streptomycin, kanamycin A or tobramycin, showed a cross-reactivity of <0.1% [12]. The only substances that have been reported as definitively interfering agents are hemoglobin, triglyceride, and bilirubin [12]. Since the assay used in the present study was based on a bacterial enzyme, the interference by TXA is unlikely. However, the issue should be studied. In the present study, the released gentamicin was biologically active after the addition of TXA to the FCS. The ZOI test results clearly show a bacterium-free zone for specimens that had been immersed in FCS + TXA. Therefore, it can be concluded that TXA does not interfere with the gentamicin elution. The resulting thickness of ZOI was about the same in all the test specimens, which indicates that the antibacterial activity of gentamicin was far beyond the minimum inhibitory concentration of the strain used. The standards for the determination of the mechanical properties of PMMA bone cements stipulate that the test specimens should have been cured for at least 1 d at 23 °C in a dry state [11,12]. The influence of fluids and eluted antibiotic on the mechanical properties of the cement is not addressed in these standards [11]. However, the influence of test fluids on the mechanical properties of the PMMA bone cement has been the subject of several studies [22,23,24,25]. Nottrott et al. reported that the increased fluid content leads to the decreased compressive modulus, tensile strength, and fatigue life of a cement, with the depreciation of each of these mechanical properties due to the intake of the test fluid into the cement specimens [23,24]. Lee et al. found a decrease in the compressive strength of bone cement (by 8–16%) and also ascribed this to the intake of the test fluid into the cement specimens [25]. Changes in the mechanical properties of the cured PMMA bone cements over time are of clinical importance and play a critical role in implant outcomes, such as implant loosening, which are often related to cement failure. In the present study, after 7 d of immersion in the test fluid, we found that the addition of TXA to FCS did not produce a significant decrease in the compressive strength of the cement. This finding is consistent with those reported by Nottrott et al., who found that a drop in the compressive strength of bone cements did not occur until 7 d after immersion of the specimens in a fluid. However, thereafter, the strength decreased by about 5.5% until 3 months of immersion [23]. In addition, it is known that the water uptake has a plasticizing effect in lowering the compressive strength and modulus of a cement after the first week of immersion of the test specimens [26]. The present compressive strength results could also be caused by the ongoing polymerization during the immersion period in the test fluid, an issue that should be the subject of a future study.

The current practice is to mix and deliver the cement using a vacuum method [3]. A conclusion on the current results is limited by the fact that the cement specimens were prepared at atmospheric pressure. Moreover, the different mixing methods affect the compressive strength and antibiotic elution. While the compressive strength is improved by vacuum mixing [27], the effect of vacuum mixing on the antibiotic elution remains conflicting, since it depends on the cement used [28].

The current study investigated the influence of a low dose (1 g) topical use of TXA. However, higher doses of topical TXA (2 and 3 g) are used to further decrease the postoperative blood loss [29]. TXA has been reported to promote the formation of St. aureus biofilms in vitro [30]. Zhang et al. reported higher amounts of bacteria on implants after a continuous TXA application compared to a single application in a mouse model.

Moreover, it is worth noting the possible effects of TXA on the periarticular and articular tissues. Wagenbrenner et al. reported no significant effects of the treatment with varying concentrations of TXA on the chondrocytes and osteogenically differentiated human mesenchymal stromal cells. In addition, concentrations up to 20 mg/mL are considered a safe limit for the topical use of TXA [31]. Further studies are needed to investigate whether a high dose of topical TXA can be safely applied to TJA, without having an influence on antibiotic efficacy and compressive strength.

## 5. Conclusions

The present study demonstrated that the addition of TXA to FCS, after immersion of the specimens for 7 d in the test solution, does not degrade any of the following properties of an ALBC brand (PALACOS R + G): Concentration of the released gentamicin, activity of the released gentamicin against a clinical isolate of *S. aureus*, and compressive strength. These results provide support for the clinical practice of intra-articular topical application of TXA into the joint space, used in a cemented TJA.

## Data Availability

The datasets used and/or analyzed during the current study are available from the corresponding author on reasonable request.
